# Generation and characterization of induced pluripotent stem cells from breast cancer patients carrying ATM mutations

**DOI:** 10.1016/j.scr.2023.103246

**Published:** 2023-11-06

**Authors:** Mao Zhang, Ravichandra Venkateshappa, Audrey Li, Michael B. Fowler, Melinda L. Telli, Joseph C. Wu

**Affiliations:** a Stanford Cardiovascular Institute, Stanford University School of Medicine, Stanford, CA 94305, USA; b Greenstone Bioscience, Palo Alto, CA 94304, USA; c Department of Medicine, Division of Cardiovascular Medicine, Stanford University School of Medicine, Stanford, CA 94305, USA; d Department of Medicine, Division of Medical Oncology, Stanford University, Stanford, CA 94305, USA

## Abstract

We generated two induced pluripotent stem cell (iPSC) lines from peripheral blood mononuclear cells (PBMCs) of breast cancer patients carrying germline ATM mutations, a gene associated with a 7% prevalence in breast cancer. These iPSC lines displayed typical morphology, expressed pluripotency markers, maintained a stable karyotype, and retained the ability to differentiate into the three germ layers. These patient-specific iPSC lines hold great potential for mechanistic investigations and the development of drug screening strategies aimed at addressing ATM-related cancer.

**Resource Table:** 

Unique stem cell lines identifier	1. SCVIi083-A 2. SCVIi084-A
Alternative name(s) of stem cell lines	1. SCVI2655 (SCVIi083-A)
	2. SCVI2591 (SCVIi084-A)
Institution	Stanford Cardiovascular Institute, Stanford, CA, US
Contact information of distributor	Joseph C. Wu, joewu@stanford.edu
Type of cell lines	iPSC
Origin	Human
Additional origin info required	Age: 72 (SCVIi083-A) and 23 (SCVIi084-A)
*for human ESC or iPSC*	Sex: Female Ethnicity if known: White (SCVIi083-A); East Asian (SCVIi084-A)
Cell Source	PBMCs
Clonality	Clonal
Method of reprogramming	Nonintegrating Sendai virus expression of human OCT4, SOX2, KLF4, and c-MYC
Genetic Modification	YES
Type of Genetic Modification	Spontaneous/naturally occurred mutation
Evidence of the reprogramming transgene loss (including genomic copy if applicable)	*RT-/q-PCR*
Associated disease	Breast Cancer
Gene/locus	Chromosome location: 11q22.3 SCVIi083-A: GRCh38: 11:108289006–108289007 SCVIi084-A: GRCh38: 11:108307919–108307919
Date archived/stock date	SCVIi083-A: 07/07/2022; SCVIi084-A: 05/25/2022
Cell line repository/bank	https://hpscreg.eu/cell-line/SCVIi083-A https://hpscreg.eu/cell-line/SCVIi084-A
Ethical approval	The generation of these iPSC lines was approved by the Administrative Panel on Human Subjects Research under Institutional Review Board (IRB) #29904 “Derivation of Human Induced Pluripotent Stem Cells (Biorepository)”

## 1. Resource utility

Germline mutations in the ATM gene are known to cause the autosomal recessive Ataxia Telangiectasia disease and also confer an increased risk of developing breast cancer. Generating iPSC lines carrying ATM variants provide an unlimited source for disease modeling, gene therapy, and screening of compounds for potential therapeutic effects (Table 1).

## 2. Resource details

The Ataxia Telangiectasia Mutated (ATM) gene, also known as ATM serine/threonine kinase, is a crucial tumor suppressor gene that belongs to the phosphatidylinositol 3-kinase-related protein kinase (PIKK) superfamily. It plays a pivotal role in DNA repair and cell cycle control (Moslemi et al., 2021). Mutations in the ATM gene are the primary cause of Ataxia Telangiectasia, an autosomal recessive neurodegenerative disorder. Recent studies have revealed a significant association between ATM variants and the risk of multiple types of cancers, particularly breast cancer (Stucci et al., 2021). Carriers of pathogenic ATM variants have a 2 to 4-fold increased risk of developing breast cancer (McDuff et al., 2021), especially early-onset cancer and bilateral breast cancer (Renwick et al., 2006). To understand these genetic associations, we successfully generated and characterized iPSC lines derived from female donors carrying specific ATM variants: c.4143dup and c.5697C > A, respectively. These iPSC lines serve as renewable and genetically relevant cellular models for investigating disease pathology and conducting drug screening for precision medicine.

**Fig. 1.**
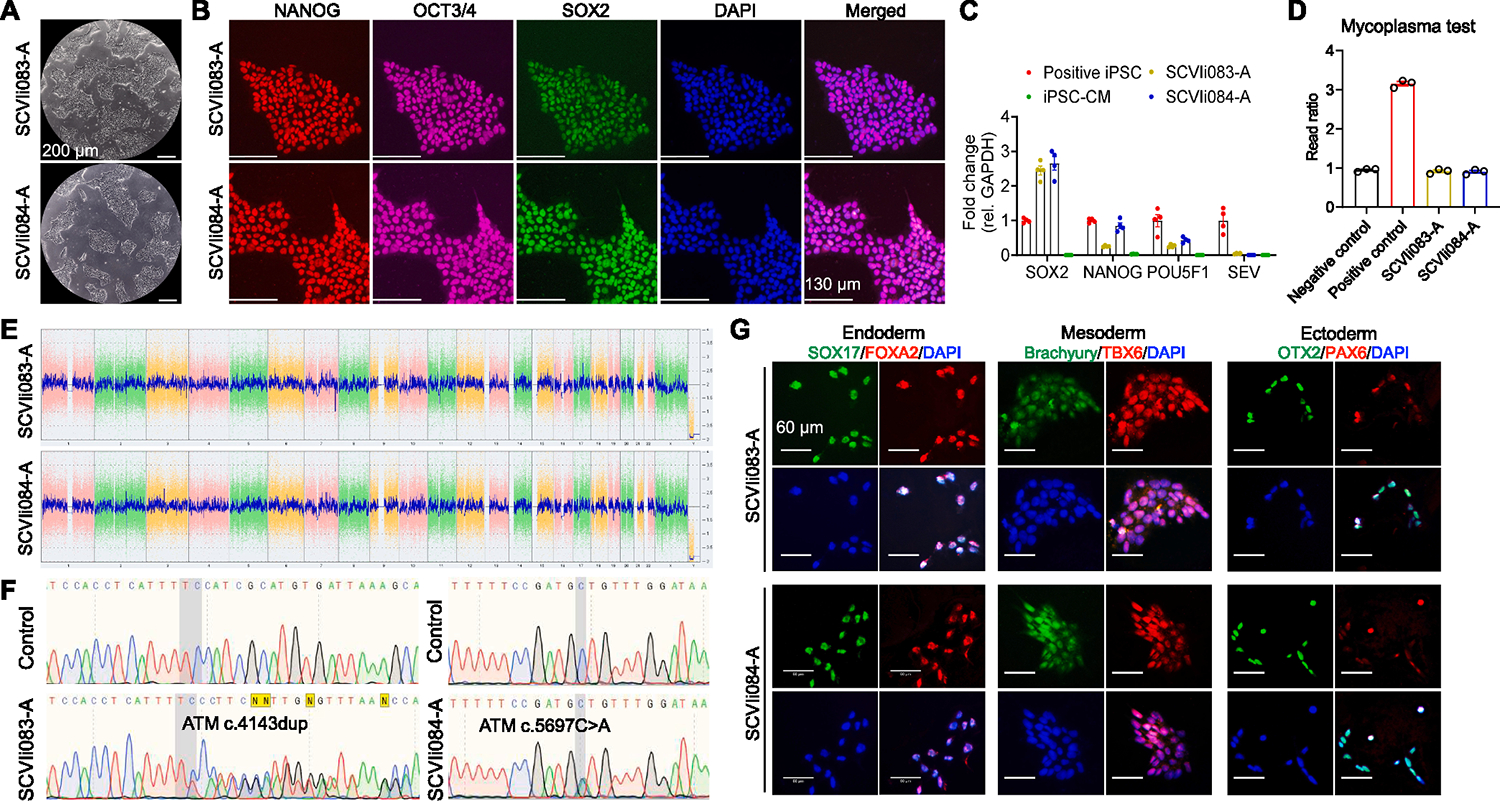
Characterization of two iPSC lines derived from breast cancer patients carrying ATM mutations.

We recruited two breast cancer patients, a 72-year-old White female who developed breast cancer, stage II (T2N1M0), of the left breast and a 23-year-old East Asian female who developed breast cancer, stage IV (T3N3M1), of the left breast. Genetic testing demonstrated that they carried the pathogenic variants *ATM* c.4143dup (ClinVar ID: 181880) and c.5697C > A (ClinVar ID: 421488), respectively. Using a Sendai Virus-based vector carrying the Yamanaka factors *OCT4, SOX2, KLF4*, and *c-MYC* (Mondéjar-Parreño et al., 2021), we successfully generated iPSCs from the patients’ peripheral blood mononuclear cells (PBMCs), named SCVIi083-A and SCVIi084-A. Both lines exhibited typical stem cell morphology when observed under bright field microscope (Fig. 1A). These cells expressed pluripotent markers (SOX2, NANOG, and POU5F1) and lost the expression of Sendai virus vector (SEV), as shown by quantitative RT-PCR (Fig. 1C). The iPSCs were further analyzed for pluripotency markers using immunofluorescence staining (Fig. 1B). The iPSC lines tested negative for mycoplasma (Fig. 1D). Karyotype analysis exhibited normal female chromosomes (Fig. 1E). Sanger sequencing demonstrated a heterozygous mutation of c.4143dup in SCVIi083-A and c.5697(C > A) in SCVIi084-A (Fig. 1F). Short tandem repeat (STR) analysis of the parental PBMCs and derived iPSCs confirmed clonal identity (Submitted in the archive with journal). Both lines were successfully differentiated into the three germ layers (Fig. 1G).

## 3. Materials and methods

### 3.1. Generation of human induced pluripotent stem cells (iPSCs)

PBMCs were isolated from the patient’s whole blood samples by Percoll gradient separation, purified by multiple washes in DPBS, and cultured in the StemPro^®^−34 SFM medium (100 ng/mL SCF, 100 ng/ mL FLT3, 20 ng/mL IL-3, 20 ng/mL IL-6, and 20 ng/mL EPO (ThermoFisher Scientific). The CytoTune^™^-iPS 2.0 Sendai Reprogramming Kit (ThermoFisher Scientific) was used for reprogramming the PBMCs following the manufacturer’s instructions.

### 3.2. Cell culture

The iPSCs were passaged at 90 % confluency using Gentle Cell Dissociation Reagent (STEMCELL^™^ technologies). Detached cells were resuspended in Brew medium with 5 μM ROCK1 inhibitor (SelleckChem), and replated onto Matrigel-coated (1:500) 6-well plates. Cells were cultured for 24 hr at 37 °C with 5 % CO_2_, and after that the media was replaced with Brew medium every two days.

### 3.3. RNA extraction and RT-qPCR

Total RNA was extracted from iPSCs at passage 13 using the miRNeasy Micro Kit (Qiagen), then cDNA was synthesized using the iScript^™^ Reverse Transcription Supermix (BIO-RAD). Target genes were examined using the TagMan^™^ Universal PCR Master Mix (ThermoFisher Scientific) and probes (Table 2).

### 3.4. Immunofluorescence staining

Cells at passage 18 were fixed with 4 % paraformaldehyde for 20 min. After two washes with DPBS, the cells were permeabilized using 0.1 % Triton X 100 in DPBS for 10 min. Subsequently, blocking was performed with 10 % goat serum in DPBS for 1 hr. Cells were then incubated with primary antibodies (Table 2) overnight at 4 °C. On day 2, the cells were washed with DPBS and incubated with the corresponding secondary antibodies (Table 2) for 1 hr at room temperature. The nuclei were counterstained with NucBlue Probes (ThermoFisher Scientific).

### 3.5. Karyotyping

The Karyotyping was performed on iPSCs at passage 12 using the KaryoStat^™^ assay (ThermoFisher Scientific).

### 3.6. Targeted sequencing

The genomic DNA was extracted using the QuickExtract^™^ DNA extraction solution (Biosearch Technologies). The PCR assay was performed using the PrimeSTAR GXL DNA Polymerase (Clontech) and the primers (Table 2) under the following conditions: 98 °C for 5 s 60 °C for 15 s, 72 °C for 30 s for 35 cycles. The PCR products were purified, and the sequencing was performed at Stanford Protein and Nuclear Acid (PAN) facility.

### 3.7. Mycoplasma detection

The mycoplasma test was conducted on iPSCs at passage 13 using the MycoAlertTM Detection Kit (Lonza), following the manufacturer’s instructions.

### 3.8. Trilineage differentiation

iPSCs at passage 17 were used for all three-germ layer differentiation. The StemXVivo Ectoderm kit (R & D systems) and the StemDiff^™^ Definitive Endoderm differentiation kit (STEMCELL^™^ Technologies) were used to derive the ectoderm and the endoderm, respectively. Mesoderm differentiation was induced by 6 μM CHIR-99021 (Selleck Chemicals) in RPMI media supplemented with B27 minus Insulin for 48 hr.

### 3.9. Short tandem repeat (STR) analysis

Genomic DNA was extracted using the DNeasy Blood & Tissue Kit (Qiagen). PCR and capillary electrophoresis were performed using the CLA IdentiFiler^™^ Direct PCR Amplification Kit and ABI3130xl at the Stanford PAN facility.

## Figures and Tables

**Table 1:** Characterization and validation.

Classification	Test	Result	Data

Morphology	Photography brightfield	Normal	Fig. 1 panel A
Phenotype	Qualitative analysis: Immunofluorescence staining	Positive expression of pluripotency markers: Oct3/4, Nanog, Sox2	Fig. 1 panel B
	Quantitative analysis: RT-qPCR	High expression levels of pluripotency markers (Nanog, Sox2, and Pou5f1) in iPSCs but significantly reduced in iPSC-CMs	Fig. 1 panel C
Genotype	Karyotype (G-banding) and resolution	Karyostat^™^ Assay, resolution 1–2 Mb: Normal karyotype: 46, XX	Fig. 1 panel E
Identity	Microsatellite PCR (mPCR) or	DNA profiling not performed	N/A
	STR analysis	16 loci tested, matched	Submitted in archive with journal
Mutation analysis (IF APPLICABLE)	Sequencing	Heterozygous SCVIi083-A: c.4143dup SCVIi084-A: c.5697C > A	Fig. 1 panel F
	Southern Blot OR WGS	N/A	N/A
Microbiology and virology	Mycoplasma test: Luminescence	Negative	Fig. 1 panel D
Differentiation potential	Directed differentiation of the three germ layers	Positive staining for all three germ layer markers	Fig. 1 panel G
List of recommended germ layer markers	Expression of these markers has to be demonstrated at mRNA (RT PCR) or protein (IF) levels, at least 2 markers need to be shown per germ layer	Positive expression of germ layer markers: Ectoderm: Pax6, Otx2; Endoderm: Sox17, Foxa2; Mesoderm: Brachyury, Tbx6	Fig. 1 panel G
Donor screening (OPTIONAL)	HIV 1 + 2 Hepatitis B, Hepatitis C	*N/A*	*N/A*
Genotype additional info (OPTIONAL)	Blood group genotyping	*N/A*	*N/A*
	HLA tissue typing	*N/A*	*N/A*

**Table 2:** Reagents details.

	Antibodies used for immunocytochemistry/flow-cytometry			
	Antibody	Dilution	Company Cat #	RRID

*Pluripotency Markers*	Rabbit Anti-Nanog	1:100	Proteintech Cat #14295–1-AP	RRID: AB_1607719
	Mouse IgG_2b_ κOct3/4 antibody	1:100	Santa Cruz Biotechnology Cat #sc-5279	RRID: AB_628051
	Goat IgG anti-Sox2	1:100	R and D Systems Cat #AF2018	RRID: AB_355110
*Ectoderm Markers*	Goat Anti-Otx2	1:200	R and D Systems Cat #AF1979	RRID: AB_2157172
	Rabbit Anti-Pax6	1:200	Thermo Fisher Scientific Cat #42–6600	RRID: AB_2533534
*Endoderm Markers*	Goat Anti-Sox17	1:200	R and D Systems Cat #AF1924	RRID: AB_355060
	Rabbit Anti-Foxa2	1:250	Thermo Fisher Scientific Cat #701698	RRID: AB_2576439
*Mesoderm Markers*	Goat Anti-Brachyury	1:200	R and D Systems Cat #AF2085	RRID: AB_2200235
	Rabbit Anti-Tbx6	1:200	Thermo Fisher Scientific Cat #PA5–35102	RRID: AB_2552412
*Secondary antibodies*	Alexa Fluor 647 Goat Anti-Mouse IgG_2b_	1:250	Thermo Fisher Scientific Cat #A-21242	RRID: AB_2535811
	Alexa Fluor 555 Goat Anti-Rabbit IgG (H + L)	1:500	Thermo Fisher Scientific Cat #A-21428	RRID: AB_141784
	Alexa Fluor 488 Donkey Anti-Goat IgG	1:1000	Thermo Fisher Scientific Cat #A-11055	RRID: AB_2534102
	**Primers Target**	**Size of band**	**Forward/Reverse primer (5’-3’)**	
*Sendai virus plasmid (qPCR)*	Sendai virus genome	181 bp	Mr042698800_mr (Thermo Fisher Scientific)	
*Pluripotency Markers (qPCR)*	NANOG	109 bp	Hs02387400_g1 (Thermo Fisher Scientific)	
*House-Keeping Genes (qPCR)*	POU5F1	77 bp	Hs00999632_g1 (Thermo Fisher Scientific)	
*Pluripotency Markers (qPCR)*	SOX2	86 bp	Hs04234836_s1 (Thermo Fisher Scientific)	
*House-Keeping Genes (qPCR)*	GAPDH	157 bp	Hs02786624_g1 (Thermo Fisher Scientific)	
*Genotyping*	ATM c.4143dup	303 bp	Forward: 5’- TGGAAGTTCACTGGTCTA -3’ Reverse: 5’- GTGTCACAAGATTCTGTTC - 3’	
*Genotyping*	ATM c.5697C > A	656 bp	Forward: 5’- CCACCAGAACCTTATAGC - 3’ Reverse: 5’- CACAGTATTGCCACAGAT - 3’	

## Data Availability

Data will be made available on request.
